# Therapeutic strategies for spinal muscular atrophy: the history and future perspective

**DOI:** 10.3389/fnhum.2026.1833269

**Published:** 2026-07-28

**Authors:** Jiayi Liu, Yang Liu, Annie Yuan, Ke Ning

**Affiliations:** 1 Jinfeng Laboratory, Chongqing, China; 2 Department of Neonatology, Chengdu Women’s and Children’s Center Hospital, School of Medicine, University of Electronic Science and Technology of China, Chengdu, Sichuan, China; 3 Sheffield Institute of Translational Neuroscience (SITraN), University of Sheffield, Sheffield, United Kingdom

**Keywords:** antisense oligonucleotides, classification, gene therapy, newborn screening, PTEN, SMN1, SMN2, spinal muscular atrophy

## Abstract

Spinal muscular atrophy (SMA) is a devastating autosomal recessive neuromuscular disorder characterized by progressive degeneration of lower motor neurons, leading to muscle weakness and atrophy predominantly manifesting in childhood. The disease is caused by homozygous deletion or loss-of-function mutations in the survival motor neuron 1 (SMN1) gene, with the nearly identical SMN2 gene acting as a critical modifier of disease severity. Since the initial clinical descriptions in 1891 and the identification of the causative gene in 1995, the field has witnessed transformative therapeutic progress. The FDA approval of nusinersen, an antisense oligonucleotide targeting SMN2 splicing, in 2016 marked the first disease-modifying therapy, followed by the gene replacement therapy onasemnogene abeparvovec in 2019 and the orally administered small-molecule splicing modifier risdiplam in 2020. These SMN-targeted therapies have substantially improved survival and motor outcomes, particularly when administered presymptomatically as enabled by increasingly widespread newborn screening programs. However, a significant proportion of patients, especially those with later-onset disease and adults, show limited responses, and existing therapies cannot reverse established neurodegeneration or muscle pathology. In this review, we trace the history of SMA research and therapy development, critically compare the three approved treatments, and discuss key unresolved challenges including treatment-switching criteria, management of late-diagnosed patients, and the emerging role of biomarkers such as neurofilament light chain and compound muscle action potential. We further explore non-SMN therapeutic strategies targeting PTEN, plastin 3, neurocalcin delta, myostatin, and neuroinflammation, and examine the rationale for combination approaches that integrate SMN restoration with muscle- and neuro-protective agents. Finally, we address future directions encompassing precision medicine, next-generation gene editing, and biomarker-driven trial design. While SMA has been transformed from a fatal childhood disorder to a treatable condition, a definitive cure for all patients remains the goal of ongoing and future research.

## 1 Introduction

Spinal muscular atrophy (SMA) is a rare, monogenic, autosomal recessive neuromuscular disorder characterized by degeneration of lower motor neurons and progressive muscle weakness. Although SMA most commonly presents in infancy or early childhood, milder phenotypes may manifest in adolescence or adulthood, including adult-onset SMA (type IV) (Ackermann et al., 2013). The estimated incidence of SMA is approximately 1 in 6,000–10,000 live births, with a carrier frequency of about 1 in 40–70 individuals. There are estimated 30,000–35,000 cases in the U.S. and European Union at any time (Agosto et al., 2023).

SMA is caused by homozygous deletion or loss-of-function mutations of the survival motor neuron 1 (*SMN1*) gene located on chromosome 5q13. Humans also carry a nearly identical paralog, *SMN2*, which differs from *SMN1* by a critical C-to-T transition in exon 7 that disrupts splicing enhancer elements. As a result, approximately 10–15% of *SMN2* transcripts include exon 7 and generate full-length, functional SMN protein, whereas the majority produce truncated, unstable isoforms that are rapidly degraded (Kashima and Manley, 2003; Lefebvre et al., 1995; Lorson et al., 1999). In the 1980s, a number of similar cases of SMA were reported and divided into several clinical groups in the 1980s. SMA was originally divided into two major groups: acute infantile SMAs (type 1) and chronic childhood SMAs (types II and III) (Brzustowicz et al., 1990; Pearn, 1980). All SMA patients were mapped to chromosome 5q13, also called 5q-SMA or simply referred to as “SMA” (Brzustowicz et al., 1990; Gilliam et al., 1990; Melki et al., 1990a; Melki et al., 1990b).

Currently, SMA is divided into five subtypes (see 2.2 for details) (Arnold et al., 2015). Both *SMN1* and *SMN2 genes* were identified in 1995 (Lefebvre et al., 1995). The *SMN1* gene is responsible for SMA, and the *SMN2* gene is an SMA modifier related to SMA severity. Muscle biopsy, which was the main method for diagnosing SMA before *SMN* genes were identified, is no longer performed (Arnold et al., 2015).

After the discovery of *SMN* genes, a new era of drug development for patients with SMA was started. Nusinersen was the first drug approved by the FDA in the USA in 2016 (Finkel et al., 2017). The second and third drugs, Onasemnogene Abeparvovec and Risdiplam, were subsequently approved by the FDA (Chaytow et al., 2021). Together, these therapies have substantially improved survival and motor outcomes, particularly when administered early. However, despite these advances, SMA cannot yet be considered curable, as many patients continue to experience residual motor impairment, systemic complications, and variable long-term outcomes. In this review, we argue that the success of SMN-centric therapies now exposes their inherent limits, motivating a deliberate shift toward multi-target combinatorial strategies. By systematically comparing the three approved drugs and examining non-SMN modifiers as therapeutic targets, we aim to provide a perspective that goes beyond summarizing current knowledge and instead offers a treatment-oriented, forward-looking synthesis.

## 2 SMA genetics

### 2.1 The history of SMA

The three periods of SMA research can be described as follows (Table 1): The first period, from the initial report of patients in 1891 to 1994, included chromosomal mapping of the SMA locus and classification of clinical subtypes. During this time, SMA was recognized as a distinct neuromuscular disorder and subdivided into clinical phenotypes based on age of onset and disease severity, and linkage studies ultimately localized SMA to chromosome 5q13 (Pearn, 1980; Melki et al., 1990b; Werdnig, 1891).

**Table 1:** History of SMA.

Periods	Years	Key scientific milestones	Diagnosis and treatments
1st	1891–1995	Clinical recognition of SMA; phenotypic classification; chromosome 5q linkage	Clinical diagnosis and classification
2nd	1995–2016	*SMN1*/*SMN2* discovery	Drug repositioning and drug discovery
3rd	2016-present	FDA approval of nusinersen and onasemnogene abeparvovec	New therapies and newborn screening

The second period involved the cloning of the *SMN* genes from 1995 to 2015 and introduced some new drug treatment for patients. This era was characterized by major advances in understanding the molecular genetics and pathophysiology of SMA, including the role of *SMN2* as a disease modifier, as well as the development of early therapeutic strategies aimed at increasing SMN protein levels (Lefebvre et al., 1995; Lorson et al., 1999; Feldkötter et al., 2002; Monani et al., 1999).

The third period commenced in 2016 with the regulatory approval of the first disease-modifying therapy for SMA, nusinersen, and continues to the present. This phase has been marked by the rapid expansion of therapeutic approaches, including antisense oligonucleotides, gene replacement therapy, and orally administered small-molecule splicing modifiers, fundamentally transforming the clinical management and natural history of SMA (Finkel et al., 2017; Mendell et al., 2017; Mercuri et al., 2018).

### 2.2 Classification of the SMA

Clinically, SMA is traditionally classified into five subtypes (types 0–IV) based on age at symptom onset and the highest motor function achieved (Table 2) (Al Dakhoul, 2017; Zappata et al., 1996; Zerres and Rudnik-Schöneborn, 1995). Type 0 is the most severe form, with prenatal onset and severe symptoms right after birth. Type I SMA (Werdnig–Hoffmann disease) is the most common and severe postnatal form, with symptom onset within the first 6 months of life and, in the absence of treatment, death or permanent ventilator dependence typically occurring within the first 2 years. Type II SMA has an intermediate phenotype, with onset between 6 and 18 months of age; affected children are able to sit independently but do not achieve independent standing or ambulation.

**Table 2:** Classification of SMA.

Type	Onset	Key clinical and molecular features	Median survival	*SMN2* copy number
0	In utero	Wide motor and sensory neuronal loss; respiratory and cardiac failure at birth	Weeks	1
I	<6 months	Neonatal hypotonia; poor feeding and head control; respiratory insufficiency; never sit	<2 years (untreated)	2–3
II	<18 months	Sit unsupported; never walk; respiratory muscle weakness	>25 years	3
III	>18 months	Walk unaided	Adult	3–4
IV	Adulthood	Progressive proximal weakness; lower limb predominance	Adult	≥4

Type III SMA (Kugelberg–Welander disease) is a milder childhood- or adolescent-onset form in which patients achieve independent ambulation, although progressive muscle weakness often leads to loss of walking ability later in life. Type IV SMA represents the mildest end of the clinical spectrum, with adult onset and relatively slow disease progression. While this classification system remains clinically useful, it is increasingly recognized that SMA represents a continuous phenotypic spectrum rather than discrete categories, particularly in the context of early diagnosis and treatment.

### 2.3 Genetics of the SMA

#### 2.3.1 *SMN* gene

In humans, two copies of *SMN* genes (*SMN 1 and SMN 2*) are located on chromosome 5q13 (Brzustowicz et al., 1990; Gilliam et al., 1990; Melki et al., 1990b). The two genes were cloned and characterized in 1995, establishing the molecular basis of SMA (Lefebvre et al., 1995). In a study of 229 patients with SMA (Lefebvre et al., 1995), homozygous deletion of *SMN1* was identified in 98.7% of cases, while the remaining 1.3% carried intragenic *SMN1* mutations, confirming *SMN1* as the disease-determining gene. Therefore, *SMN1* is considered to be the key gene with nine exons (exons 1, 2a, 2b, 3, 4, 5, 6, 7, and 8) (Lorson et al., 1999; Bürglen et al., 1996). The start codon (ATG) is located at exon 1, and the stop codon (TAA) is located at exon 7 (Lorson et al., 1999; Bürglen et al., 1996). The full-length SMN protein comprises 294 amino acids with an approximate molecular weight of 38 kDa (Cho and Dreyfuss, 2010; Coovert and Coovert, 1997).

#### 2.3.2 The SMN protein

The SMN protein is found in the cytoplasm (Liu and Dreyfuss, 1996), nucleolus (Charroux et al., 2000), gem and Cajal bodies in the nucleus (Carvalho et al., 1999), neuronal growth cones (Fan and Simard, 2002), and neuronal extensions (Fallini et al., 2010). The function of the SMN protein are involved in various processes, including actin cytoskeleton dynamics (Hensel and Claus, 2018), mRNA transport (Donlin-Asp et al., 2016), bioenergetics pathways (Boyd et al., 2017), RNA metabolism (Li et al., 2003), ubiquitin homeostasis (Groen and Gillingwater, 2015), and synaptic vesicle release (Kong et al., 2009) (Table 3), which are possibly associated with part of the underlying mechanism of SMA pathophysiology. The function of the SMN protein is also related to the splicing of pre-mRNAs or snRNP biogenesis (Fischer et al., 1997; Liu et al., 1997; Monani, 2005). Burghes and Beattie suggested that a decrease in the SMN protein level during snRNP assembly causes gene splicing, and the disruption of mRNA transport in the SMN protein in neurons results in SMA (Burghes and Beattie, 2009). Additionally, the specific binding of SMN protein domains to their partner proteins is important for their function (Hebert et al., 2001; Martin et al., 2012; Singh et al., 2017). The SMN protein is associated with a wide range of cellular functions, including the ubiquitin–proteasome system, pre-mRNAs splicing, endocytosis, cytoskeletal dynamics, autophagy, mRNA trafficking, mitochondrial dysfunction and bioenergetic pathways (Singh et al., 2017; Chaytow et al., 2018).

**Table 3:** Localization and function of SMN protein in neuronal cells.

Location	Function
Nucleolus	Ribosome biogenesis
Gems and Cajal bodies	snRNP biogenesis, maturation, and recycling
Cytoplasm	snRNP biogenesis, actin dynamics, ubiquitin homeostasis, and bioenergetics pathways
Axons	mRNA transport
Synapse	Actin dynamics and vesicle release

Consistent with these findings, SMN deficiency disrupts multiple cellular pathways, including the ubiquitin–proteasome system, pre-mRNA splicing, endocytosis, cytoskeletal dynamics, autophagy, mRNA trafficking, and mitochondrial bioenergetics, underscoring the multi-systemic cellular consequences of SMN loss in SMA (Martin et al., 2012; Tripsianes et al., 2011).

#### 2.3.3 SMA pathology

SMA pathology is associated with skeletal muscle, neuromuscular junctions (NMJs) and lower motor neurons. A complex of SMN proteins and the heterogeneous nuclear ribonucleoprotein R (hnRNP R) is important for the function of motor neurons, particularly in the interaction with *β*-actin mRNA and its translocation to axons and growth cones, as well as in axonal RNA metabolism or splicing regulation (Fallini et al., 2012; Rossoll et al., 2003). In the NMJs, AChR cluster formation and synaptic transmission are impaired in SMA mouse models (Ackermann et al., 2013; Kariya et al., 2008; Riessland et al., 2017). Additionally, overexpression of plastin 3 protein (PLS3) increases survival and improves motor function by restoring impaired neurotransmission and addressing NMJ dysfunction in severe SMA model mice (Hosseinibarkooie et al., 2016). Furthermore, neurocalcin delta (NCALD) suppression may have a protective effect on SMAs (Riessland et al., 2017) and the disruption of the SNARE complex, which is associated with SMN, dysregulates neurotransmission at NMJs (Bottai and Adami, 2013).

Skeletal muscle pathology also contributes directly to SMA disease progression. Selective depletion of SMN in skeletal muscle leads to muscle fiber atrophy, mitochondrial abnormalities, and increased susceptibility to apoptosis, demonstrating that SMN deficiency exerts both cell-autonomous and non–cell-autonomous effects in muscle tissue (Bottai and Adami, 2013; Kim et al., 2020). Moreover, SMN depletion disrupts mitochondrial homeostasis and bioenergetic function in muscle and motor neurons, promoting oxidative stress and cell death pathways (Ikenaka et al., 2023).

## 3 SMN2

The molecular mechanisms governing *SMN2* exon 7 splicing and gene expression have been extensively dissected over the past three decades, revealing a complex regulatory network that operates at multiple levels—from cis-acting splicing elements and trans-acting splicing factors to epigenetic modifications of the chromatin landscape. A comprehensive overview of these regulatory layers, including the roles of histone acetylation, DNA methylation, and their implications for therapeutic targeting of *SMN2*, has recently been provided by Virata and colleagues (Li et al., 2025). The following subsections summarize the key genetic and molecular features of *SMN2* that underpin its function as the primary disease modifier in SMA, and which have directly informed the design of the splicing-modifying therapies discussed later in this review.

### 3.1 The gene

The *SMN1* gene is located at the telomeric side, while the *SMN2* gene is at the centromeric side and has an almost identical sequence (C-to-T) with only a few nucleotide differences in the entire gene. A key difference for *SMN2* is the single-nucleotide difference (C-to-T) at the sixth nucleotide position in exon 7 (Lefebvre et al., 1995; Monani et al., 1999). As a consequence, approximately 10–15% of *SMN2* transcripts generate full-length, functional SMN protein, while the majority produce exon 7–skipped transcripts encoding unstable truncated isoforms (Lefebvre et al., 1995; Monani et al., 1999; Cho and Dreyfuss, 2010). Complete loss of SMN is embryonically lethal in mice; however, introduction of the human *SMN2* gene rescues embryonic viability and generates a severe SMA-like phenotype, demonstrating that *SMN2* is sufficient to support development but not normal motor neuron maintenance (Hsieh-Li et al., 2000; Monani, 2000).

### 3.2 Splicing

The C-to-T transition in exon 7 disrupts splicing regulatory elements and profoundly alters *SMN2* pre-mRNA processing, resulting in predominant skipping of exon 7 and markedly reduced production of full-length SMN protein (Kashima and Manley, 2003; Lorson et al., 1999). Although full-length *SMN2* transcripts are produced, they constitute only a minor fraction of total *SMN2* mRNA (Coovert and Coovert, 1997; Lefebvre et al., 1997). Experimental evidence indicates that this nucleotide substitution disrupts an exonic splicing enhancer (ESE) motif required for binding of the serine/arginine-rich splicing factor SF2/ASF (SRSF1), thereby altering recruitment of the U2 small nuclear ribonucleoprotein complex and U2AF to the 3′ splice site (Kashima and Manley, 2003; Cartegni et al., 2006; Rio, 1993). This imbalance between positive and negative splicing regulators shifts exon 7 toward exclusion, establishing the molecular basis of SMN deficiency in SMA (Singh et al., 2004a). The ∆7-SMN protein does not function properly and has difficulty oligomerizing and forming a multimeric protein complex, which is associated with the severity of SMA (Lorson et al., 1998), and is degraded two times faster than the full-length SMN protein by the ubiquitin proteasome system (Burnett et al., 2009).

### 3.3 Severity and copy number of *SMN2*

Disease severity in SMA is strongly modified by *SMN2* copy number. Patients with milder phenotypes (types II–IV) generally harbor more copies of *SMN2* than those with severe type I disease (Feldkötter et al., 2002; McAndrew et al., 1997; Wirth et al., 2006). Increased *SMN2* copy number partially compensates for loss of *SMN1* by elevating residual SMN protein levels and thereby ameliorating clinical severity (Lefebvre et al., 1997).

The genotype–phenotype correlation is commonly summarized as follows: one *SMN2* copy is typically associated with type 0; two to three copies with type I; three copies with type II; three to four copies with type III; and four or more copies with type IV disease (Wirth et al., 2006; Harada et al., 2002). However, this relationship is not absolute. Phenotypic variability occurs, particularly in individuals with intragenic *SMN1* mutations or additional genetic modifiers, indicating that *SMN2* copy number alone does not fully determine clinical outcome (Riessland et al., 2017; Mailman et al., 2002; Oprea et al., 2008).

### 3.4 SMA phenotype and other genetic modifiers

In addition to *SMN2* copy number, disease severity in SMA is significantly influenced by additional genetic modifiers. *PLS3* was first identified as a protective modifier in a family-based study, in which asymptomatic female carriers of *SMN1* deletions exhibited markedly elevated *PLS3* expression compared with affected siblings (Oprea et al., 2008). Increasing PLS3 has been shown to promote axon elongation in motor neurons and rescues neuromuscular junction integrity, thereby improving motor function and survival in cellular and animal models of SMA (Ackermann et al., 2013; Hosseinibarkooie et al., 2016). Neurocalcin delta (*NCALD*) has been identified as another major disease modifier. Reduced expression of *NCALD* correlates with milder SMA phenotypes in human patients, and genetic or antisense-mediated suppression of *NCALD* significantly improves motor neuron survival, neuromuscular transmission, and overall disease progression in SMA mouse models. Genome-wide linkage and transcriptomic analyses further support *NCALD* as a robust modifier of SMA severity, independent of *SMN2* copy number (Riessland et al., 2017).

Together, these findings demonstrate that SMA phenotype reflects a complex genetic network in which *SMN2* dosage interacts with additional modifiers that regulate cytoskeletal dynamics, synaptic stability, and neuronal vulnerability, providing a strong biological rationale for combinatorial therapeutic strategies beyond SMN restoration alone.

### 3.5 Epigenetic regulation of SMN2 expression

The molecular mechanisms governing *SMN2* exon 7 splicing and gene expression have been extensively dissected over the past three decades, revealing a complex regulatory network that operates at multiple levels—from cis-acting splicing elements and trans-acting splicing factors to epigenetic modifications of the chromatin landscape. A comprehensive overview of these regulatory layers, including the roles of histone acetylation, DNA methylation, and their implications for therapeutic targeting of *SMN2*, has recently been provided by Virata and colleagues (Virata et al., 2025).

Beyond the well-characterized cis-acting splicing elements and trans-acting splicing factors, *SMN2* expression is also subject to epigenetic modulation at both the transcriptional and post-transcriptional levels.

Histone acetylation status at the *SMN2* locus plays a critical role in regulating gene transcription. Histone deacetylase inhibitors (HDACi), such as valproic acid (VPA), phenylbutyrate, and trichostatin A, have been shown to increase *SMN2* transcription by promoting a more open chromatin conformation, thereby elevating SMN protein levels in patient-derived cells (Brichta et al., 2003; Kernochan et al., 2005; Sumner et al., 2003). Kernochan and colleagues demonstrated that the *SMN* gene has a reproducible pattern of histone acetylation that is largely conserved among different tissues and species, and that pharmacological manipulation of this epigenetic determinant is feasible (Kernochan et al., 2005). However, as discussed in Section 4.5, clinical translation of HDACi monotherapy in SMA has yielded inconsistent results, highlighting the gap between transcriptional upregulation and meaningful disease modification (Kissel et al., 2011; Swoboda et al., 2009).

In parallel, DNA methylation of the *SMN2* promoter region has emerged as another layer of regulatory complexity. Hauke and colleagues demonstrated that the *SMN2* gene is subject to gene silencing by DNA methylation, containing four CpG islands with highly conserved methylation patterns (Hauke et al., 2009). Their comprehensive analysis of *SMN2* methylation in patients with severe versus mild SMA carrying identical *SMN2* copy numbers revealed a correlation of CpG methylation at positions −290 and −296 with disease severity and the activity of the first transcriptional start site of *SMN2* (Hauke et al., 2009). Furthermore, they showed that the methyl-CpG-binding protein 2 (MeCP2), a transcriptional repressor, binds to the critical *SMN2* promoter region in a methylation-dependent manner, and that certain HDAC inhibitors—including vorinostat and romidepsin—can bypass *SMN2* gene silencing by DNA methylation (Hauke et al., 2009). Zheleznyakova and colleagues performed the first genome-wide methylation profiling of SMA patients and healthy individuals, identifying strong significant differences in methylation levels between SMA patients and healthy controls at CpG sites close to genes associated with Rab and Rho GTPase activity—pathways critical for vesicle formation, actin dynamics, and axonogenesis (Zheleznyakova et al., 2013).

More recently, Zwartkruis and colleagues conducted a comprehensive analysis of methylation patterns across the 30 kb *SMN2* gene using native long-read nanopore and targeted short-read bisulfite sequencing (Zwartkruis et al., 2025). Their long-read analysis of 29 SMA patients identified tissue-specific variation in *SMN2* intronic regions and the 3′UTR, while further analysis of blood-derived DNA from 365 SMA patients found no association between *SMN2* methylation and disease severity or treatment response, excluding blood-derived DNA methylation as a predictive biomarker. However, they discovered significant age-associated variation in *SMN2* methylation in intron 1 and the 3′UTR, suggesting a possible role in modifying SMN expression during development and aging (Zwartkruis et al., 2025).

Collectively, these findings indicate that epigenetic mechanisms—particularly histone acetylation and DNA methylation—contribute to the regulation of *SMN2* expression and may influence phenotypic variability in SMA, independent of *SMN2* copy number. They also raise the possibility that baseline epigenetic status could modulate individual responses to splicing-targeted therapies such as nusinersen and risdiplam, although this hypothesis awaits prospective clinical validation.

## 4 Drug development

### 4.1 Therapeutic choices

As the *SMN2* copy number can affect disease severity, targeting *SMN2* may provide a potential solution for SMA. Early proof-of-concept studies demonstrated that increased expression of SMN protein is sufficient to rescue motor neuron degeneration and extend survival in severe SMA mouse models (Hsieh-Li et al., 2000; Monani, 2000). Consequently, an increasing number of researchers believe that increasing the production of full-length *SMNs* would be beneficial to patients with SMA. The *SMN1* and *SMN2* genes were first reported in 1995 (Lefebvre et al., 1995) and many studies were conducted from 1995 to 2016 to identify a cure for SMA. This work ultimately led to the approval of nusinersen in 2016, the first disease-modifying therapy for SMA (Finkel et al., 2017).

### 4.2 Targets for *SMN2* splicing

The alternative splicing of *SMN2* exon 7 is regulated by multiple cis-acting elements and trans-acting splicing factors, providing several therapeutic entry points. Two targets, exon-specific splicing enhancer (ESSENCE) and targeted oligonucleotide enhancer of splicing (TOES), are associated with *SMN2* splicing (Cartegni and Krainer, 2003; Skordis et al., 2003) and target exon-specific splicing enhancement (ESE) in *SMN2* exon 7. The ESSENCE compound has 10 arginine/serine (RS) repeat regions and can emulate the ESE-dependent function of SR proteins, as well as rescuing *SMN2* splicing (Cartegni and Krainer, 2003). The TOES compound is a bifunctional oligonucleotide composed of a GGA-repeat domain that recruits splicing activator complexes and an antisense domain that tethers the activator to the exon 7 region (Skordis et al., 2003). Although these synthetic enhancer approaches provided important proof-of-concept, their clinical translation has been limited by delivery and specificity challenges. Instead, therapeutic development shifted toward targeting negative regulatory elements.

In parallel, studies of intronic splicing silencers (ISS) revealed that suppression of inhibitory elements within intron 7 strongly promotes exon 7 inclusion. In particular, ISS-N1 located downstream of exon 7 functions as a dominant negative regulator of *SMN2* splicing (Singh et al., 2006). Antisense oligonucleotides targeting ISS-N1 effectively restore exon 7 inclusion, increase SMN protein levels, and dramatically improve survival and neuromuscular function in SMA mouse models (Hua et al., 2011; Hua et al., 2008). Systematic mapping of splicing regulatory elements within the flanking introns has further refined these targets and guided therapeutic oligonucleotide design (Singh et al., 2004b).

### 4.3 Cellular and animal models

Due to the limitations of animal models, it is difficult to predict whether the effects observed can be replicated in humans. Recent stem cell research has opened new ways to directly observe the pathophysiological mechanisms in neurons derived from iPSCs (Ebert et al., 2009). Beyond motor neurons, iPSCs have been differentiated into multiple non-neuronal cell types relevant to SMA systemic pathology, including cardiomyocytes, endothelial cells, and pancreatic cells, enabling investigation of the multi-organ consequences of SMN deficiency (De Vos et al., 2016; Lippmann et al., 2014; Mutsaers et al., 2011; Sun et al., 2022). iPSC-derived motor neurons from different types of SMA patients have allowed researchers to explore the disease mechanism at the cellular and subcellular level. This has led to the development of different therapeutic targets for SMA (Bowerman et al., 2017) (Table 4).

**Table 4:** Models for non-SMN targets.

Models	Effect on non-SMN molecular targets or drug candidate
iPSC-derived motor neurons	SMN expression, target expression, axonal length, and branching
*In vitro* BBB model	Ability to cross BBB, SMN expression, and target expression
Zebrafish SMA models	Axon growth and branching, survival, and motor function
Mouse models	Survival, weight, SMN expression, target expression, neuromuscular function, neuromuscular pathology, and behaviors

Other cellular and animal models have also been used to understand the pathogenesis of SMA and to develop new therapies for SMA. In the past two decades, various mouse models have been developed to predict different disease severity ranges. Since a complete knockout (*SMN−/−*) is embryonically lethal, alternative SMA mouse models have been developed by genetic modifications (Monani, 2000; Zhou et al., 2015). Although these limitations are unavoidable, animal models are still very important systems for understanding the disease process and for evaluating potential therapeutic approaches (Table 4).

### 4.4 Gene therapy for SMA

#### 4.4.1 Lentiviral vectors

The first proof of concept in gene therapy for SMA was reported in 2004. A lentivector expressing SMN was constructed and injected into multiple muscles in a mouse model on postnatal day, suggesting that overexpression of the exogenous *SMN* gene could be an option for treating SMA through retrograde axonal transport (Azzouz et al., 2004).

#### 4.4.2 AAV vector

A major breakthrough occurred in 2009 when intravenous administration of self-complementary adeno-associated virus serotype 9 (scAAV9) was shown to efficiently cross the blood–brain barrier and transduce widespread neuronal and glial populations throughout the central nervous system (Foust et al., 2009).

In 2010, we reported similar results by injecting scAAV9-coSMN into the facial vein on postnatal day 1 and rescuing the SMA phenotype in a severe mouse model (Valori et al., 2010). Systemic delivery of scAAV9 encoding codon-optimized SMN into neonatal SMA mice produced robust and sustained rescue of motor function, prolonged survival, and preservation of motor neurons, thereby establishing AAV9-mediated gene therapy as a highly effective therapeutic strategy for SMA.

#### 4.4.3 Non-SMN targets emerging from SMA pathology

SMN-dependent gene therapies are more likely to be effective when they are administered as early as possible, even presymptomatically (Valori et al., 2010; Bevan et al., 2010). However, they do not completely restore normal neuromuscular physiology in most patients, and disease progression may continue in treated individuals. This recognition has motivated the identification of non-SMN targets that contribute to motor neuron vulnerability, neuromuscular junction integrity, and muscle maintenance. Among the earliest studied pathways was the RhoA-ROCK cascade, whose inhibition improved survival in an intermediate SMA mouse model (Bowerman et al., 2010; Luo et al., 1997). Subsequent work has highlighted additional modifiers with therapeutic potential, including the endoplasmic reticulum protein stasimon (Bowerman et al., 2017), cyclin-dependent kinase 5 (Cdk5) (Doktor et al., 2017; Miller et al., 2023), and the ubiquitin-like modifier activating enzyme Uba1 (Groen and Gillingwater, 2015; Foust et al., 2009). Three genetic modifiers—PTEN, PLS3, and NCALD—have attracted particular attention because of their direct links to SMA severity in patients and because their modulation has shown preclinical efficacy, making them candidate targets for combination therapy.

#### 4.4.4 Therapeutic rationale for PTEN, PLS3, and NCALD as adjunctive targets

Phosphatase and tensin homolog (PTEN) acts as a negative regulator of the PI3K/AKT/mTOR signaling pathway, which promotes axonal growth and neuronal survival. In SMA, SMN deficiency leads to elevated PTEN protein levels, thereby suppressing AKT activity and downstream pro-growth signals. PTEN knockdown in SMN-deficient motor neurons restores axon elongation and improves survival in vitro (Ning et al., 2010). As a therapeutic strategy, PTEN inhibition offers a neuroprotective mechanism that is independent of SMN restoration, and it may be particularly relevant for patients in whom motor neurons are already compromised. To date, no clinical trial has evaluated a PTEN-targeted intervention in SMA; systemic delivery of PTEN-directed antisense oligonucleotides or small-molecule inhibitors remains at the preclinical stage, with safety and selectivity challenges to be addressed.

Plastin 3 (PLS3) encodes an F-actin-bundling protein involved in endocytosis and cytoskeletal dynamics. PLS3 was identified as a protective modifier in asymptomatic female carriers of SMN1 deletions who exhibited high PLS3 expression (Mailman et al., 2002). Overexpression of PLS3 in SMA mouse models rescues neuromuscular junction integrity, restores synaptic transmission, and extends survival, even in the presence of very low SMN levels (Ackermann et al., 2013; Hosseinibarkooie et al., 2016). The mechanism involves delayed axon pruning and improved endocytic trafficking. Adeno-associated virus-mediated PLS3 delivery has been proposed as a gene therapy approach independent of SMN, but preclinical evaluation has not yet advanced to clinical trials. PLS3 augmentation may be best suited as an adjunct to SMN-targeted therapy, particularly to protect the distal neuromuscular axis.

Neurocalcin delta (NCALD) is a calcium-binding protein that was identified as a negative regulator of endocytosis and neuronal growth. Reduced NCALD expression correlates with milder SMA phenotypes in humans, and antisense-mediated NCALD suppression improves motor neuron survival, neuromuscular transmission, and overall disease progression in SMA mouse models (Riessland et al., 2017; Torres-Benito et al., 2019). Notably, when combined with nusinersen, NCALD antisense oligonucleotide therapy further ameliorated the phenotype in mice, providing a direct preclinical demonstration of a combinatorial approach. This positions NCALD as a promising adjunctive target; however, translation to human studies has not yet been reported.

Together, these three modifiers exemplify the concept that SMA severity is governed not only by SMN2 copy number but also by a network of genetic factors that regulate cytoskeletal integrity, synaptic function, and cellular survival pathways. Their therapeutic targeting—alone or in combination with SMN-enhancing drugs—represents an important frontier for future clinical development. A summary of representative non-SMN-targeted therapeutic strategies by mechanism and clinical stage is provided in Table 5.

**Table 5:** Representative non-SMN-targeted therapeutic strategies in SMA.

Therapeutic strategy	Drug/Approach	Mechanism	Clinical status	Notes
Anti-myostatin	Apitegromab	Inhibits promyostatin activation	Phase 2 completed	Synergy with nusinersen observed
Muscle contractility	Reldesemtiv	FSTA: enhances troponin Ca^2+^ sensitivity	Development discontinued	Functional gains; limited long-term benefit
Muscle growth (IGF-1)	BVS857	IGF-1 mimetic	Development discontinued	High immunogenicity
Anti-inflammatory	Nusinersen, miR-146a	Suppresses IL-6, TNF-α, NF-kB signaling	Ongoing/preclinical	Secondary benefit from SMN therapy
Autophagy/ER stress	Salubrinal, Ceapins	Modulate UPR, reduce stress-induced apoptosis	Preclinical	Effective in SMA models
AAV delivery innovation	Next-gen AAV vectors	Lower toxicity, CNS-specific tropism	Preclinical	Targets redosing and immune limitations

### 4.5 Existing medications for SMA

The search for drugs to treat SMA started with the repositioning of the existing medications, such as gabapentin, hydroxyurea, aclarubicin, riluzole, thyroid-stimulating hormone-releasing hormone (TRH), valproic acid (VPA), and salbutamol (Iannaccone and Nelson, 2017).

Aclarubicin, an anthracycline anticancer agent, and the tetracycline derivative PTK-SMA1 were among the first compounds reported to increase SMN protein expression in cellular models by modulating *SMN2* transcription and splicing (Andreassi et al., 2004; Hastings et al., 2009). Subsequent studies confirmed that aclarubicin could elevate full-length SMN levels in patient-derived fibroblasts, although systemic toxicity and limited central nervous system penetration precluded further clinical development (Andreassi et al., 2004).

Valproic acid (VPA) was found to increase the level of SMN protein by increasing the transcription of *SMN2* and correcting the splicing of *SMN2* exon 7 (Brichta et al., 2003; Sumner et al., 2003). Early open-label and small cohort studies reported modest improvements in motor function in subsets of patients with SMA (Weihl et al., 2006). However, multiple randomized controlled trials and longer-term studies subsequently failed to demonstrate consistent clinical benefit, and concerns regarding hepatotoxicity, weight gain, and teratogenicity further limited its clinical utility in SMA (Kissel et al., 2011; Swoboda et al., 2009; Swoboda et al., 2010). Collectively, these early pharmacological approaches provided important proof-of-concept for pharmacological modulation of SMN expression but underscored the limitations of non-targeted drug repurposing strategies for achieving meaningful disease modification in SMA.

### 4.6 Branaplam and other discontinued splicing modifiers

In parallel with the successful clinical development of risdiplam, another oral small-molecule splicing modifier, branaplam (LMI070, NVS-SM1), was investigated for SMA. Branaplam was originally identified through a phenotypic high-throughput screen and was found to promote *SMN2* exon 7 inclusion by stabilizing the interaction between the U1 small nuclear ribonucleoprotein and the 5′ splice site, a mechanism overlapping with that of risdiplam (Palacino et al., 2015; Ratni et al., 2018). A Phase 1/2 open-label, first-in-human, proof-of-concept study (MOONFISH; NCT02268552) was conducted in infants with Type 1 SMA who had exactly two copies of *SMN2* [ClinicalTrials.gov NCT02268552]. The study evaluated the safety, tolerability, pharmacokinetics, pharmacodynamics, and efficacy of once-weekly oral branaplam. Part 1 of the trial involved dose escalation with weekly dosing over 13 weeks, while Part 2 enrolled new patients into dose cohorts with weekly dosing for up to 52 weeks.

Despite these efforts, Novartis announced in 2021 the decision to discontinue development of branaplam for SMA. According to a community update issued by the company, this decision was driven by the rapid evolution of the SMA treatment landscape and the recognition that branaplam would not offer a sufficiently differentiated therapeutic option given the existing availability of three approved therapies [Novartis community update, 2021]. Subsequently, Novartis redirected its efforts toward developing branaplam for Huntington’s disease. In that context, branaplam was shown to lower huntingtin (HTT) transcript levels by promoting inclusion of a pseudoexon containing a premature stop codon in the HTT pre-mRNA (Keller et al., 2022).

The branaplam experience illustrates several important lessons for the SMA therapeutic landscape. It demonstrates that a favorable preliminary profile in early-phase trials does not guarantee continued development, particularly when the competitive landscape shifts rapidly. It also highlights the high bar for market entry that new SMA therapies must clear: they must offer meaningful differentiation—whether through superior efficacy, improved convenience, or a better safety profile—over existing options. While branaplam never reached regulatory approval, its development provided valuable insights into the mechanisms of *SMN2* splicing modulation and contributed to the broader understanding of this therapeutic class.

## 5 FDA-approved drugs for SMA

Before examining the three FDA-approved therapies individually, it is crucial to evaluate them through a comprehensive clinical framework with relevant dimensions. While Nusinersen, onasemnogene abeparvovec, and risdiplam all share the fundamental goal of increasing functional SMN protein levels to restore muscle function, they diverge significantly in terms of their molecular strategies administration routes, patient eligibility, safety concerns, and economic burden.

These distinctions are essential in the current therapeutic landscape because each therapy addresses only a fraction of the SMA pathological process and leave lingering questions regarding long-term durability and incomplete motor recovery. Given that these drugs are not interchangeably “equivalents,” it goes beyond a single drug selection and complexes the decision-making process for both clinicians and families. This comparison therefore reinforces the central theme of this review: although SMN-targeted therapies represent a transformative leap in SMA, targeting SMN proteins alone is likely insufficient for a definitive cure.

### 5.1 Nusinersen

Nusinersen became the first FDA-approved drug for the treatment of SMA in the US in 2016 (Table 6), after the successful achievement of the primary endpoint in the ENDEAR Phase 3 trial (Nishio et al., 2023). This approval represented a notable milestone in the management of a disease that had long been considered incurable due to the lack of effective therapeutic choices. Since ASOs are incapable of crossing the blood–brain barrier (BBB) to reach motor neurons in the central nervous system (CNS), nusinersen has to be administered through intrathecal injection (Nishio et al., 2023).

**Table 6:** FDA-approved drugs for SMA.

Characteristic	Nusinersen (Spinraza)	Onasemnogene abeparvovec (Zolgensma®)	Risdiplam (Evrysdi)
Therapeutic class	Antisense oligonucleotide	Adeno-associated virus mediated *SMN1* gene replacement therapy	Orally administered small molecular compound
Mechanism of action	Modification of *SMN2* pre-mRNA splicing; binds ISS-N1 in intron 7 to promote inclusion of exon 7	Delivers a functional copy of *SMN1* transgene through an AAV9-vector	Modification of *SMN2* pre-mRNA splicing; interacts with intron 7/exon 7 splice regulatory regions
Administration	Intrathecal injection	Intravenous	Daily oral administration
FDA approved year	2016	2019	2020
Approved age eligibility	Pediatric and adult patients	Pediatric patients younger than 2 years	Pediatric and adult patients
Clinical trials involving symptomatic patients	ENDEAR, CHERISH	START	FIREFISH, SUNFISH
Clinical trials involving pre-symptomatic patients	NURTURE	SPR1NT	RAINBOWFISH
Clinical strengths	Strong advantage when treatment begins early; positive outcomes across symptomatic and presymptomatic patients	One-time treatment; stable SMN expression; strong advantage in very early treated patients	Non-invasive strategy; broad applicability across all age groups
Clinical limitations	CNS-focused delivery lack the tissue distribution to peripheral tissues; feedback differs in different disease stages and SMN2 copy number	May require additional nutritional or respiratory support; less improvements in older or heavier patients	Necessitates long-term daily treatment; motor function recovery may reach stabilization or plateau in symptomatic patients

The therapeutic mechanism of nusinersen focus on targeting the intronic splicing silencer N1 (ISS-N1) located on intron 7, a regulatory sequence adjacent to exon 7 that was identified through systematic screening (Singh et al., 2004a). This binding of nusinersen to the +11 to +24 nucleotide region of ISS-N1 interferes with the recruitment of hnRNP A1 and A2 splicing repressors, therefore stimulating the inclusion of exon 7 in *SMN2* pre*-*mRNA and the production of full-length SMN protein (Hua et al., 2008). Given that ISS-N1 also interacts with multiple splicing factors, hnRNP A1 and A2 are unlikely to represent the sole regulators whose activity is affected by nusinersen binding. In addition to blocking repressor binding, structural remodeling of the pre-mRNA induced by nusinersen is believed to contribute to its splicing-corrective activity (Sun et al., 2022).

The efficacy of Nusinersen has been evaluated in multiple large-scale clinical trials. The Phase 3 ENDEAR trial (*n* = 122 infants) and the CHERISH trial (*n* = 126 children with Types II and III) demonstrated notable improvements in motor function compared to sham controls in symptomatic patients (Mercuri et al., 2018; Cartegni and Krainer, 2003). The primary endpoint of the NURTURE trial (*n* = 25 presymptomatic infants with Type II) was survival time and the findings implied treatment initiated during the presymptomatic period maximizes motor function gains, although outcomes vary by genotype (Torres-Benito et al., 2019). Real-world quantitative evidence further strengthens these clinical trial results, with symptomatic patients experiencing a stable increase in CHOP-INTEND motor scores over a 22-month observation period, rising from an average yield of 4.2 points to 17.8 points (Lemska et al., 2024).

Despite these encouraging advances, heterogeneity in treatment response and residual disability remain as fundamental concerns. Long-term follow-up studies show that motor skill recovery is strongly influenced by the copy number of *SMN2*. Among patients with three *SMN2* copies, 32% were able to stand independently and 14% could walk without support. In contrast, none of the patients with two *SMN2* copies attained standing independently or walking (Audic et al., 2024). In addition, nusinersen does not completely prevent the progression of certain disease manifestations. The development of early scoliosis was present in 100% of patients with two copies of *SMN2* and in 64% of those with three copies of *SMN2* after 3 years of treatment (Audic et al., 2024). While the release of nusinersen has substantially altered the natural history of SMA, uncertainties remain regarding the long-term durability of its therapeutic effects and whether sufficient SMN protein can be produced in patients with only one *SMN2* copy.

### 5.2 Onasemnogene abeparvovec

Onasemnogene abeparvovec is a gene replacement therapy designed to deliver a functional copy of the *SMN1* gene via a self-complementary adeno-associated virus serotype 9 (scAAV9) vector (Zhang et al., 2024). Approved by the FDA in 2019 and subsequently authorized in Europe and Japan in 2020, it is administered as a single intravenous infusion. The AAV vector penetrates the BBB and enters into the host cells, where the vector genome translocates into the nucleus and remains as episomal DNA, enabling a prompt and sustained expression of SMN protein (Zhang et al., 2024; Mendell et al., 2021). Gene therapy is particularly suitable for SMA treatment for several reasons: the disease can be fatal without treatment; the cause of the disease is known and specific; toxicity from gene therapy is minimal; animal models with clear SMA phenotypes are available; and the SMN cDNA length is suitable for AAV vectors (Govoni et al., 2018).

The clinical efficacy of Onasemnogene abeparvovec has been demonstrated in several studies. In the Phase 1 START trial (*n* = 12 in the high-dose cohort), 24-month follow-up data revealed a significant shift from the natural history of the disease: survival without permanent ventilation increased from only a rate of approximately 8 to 100% among treated participants (Mendell et al., 2017; Zhang et al., 2024). Clinical responses to this one-time treatment were also observed rapidly, with the CHOP INTEND score increasing by an average of 9.8 points within 1 month post injection (Mendell et al., 2017). Among the 12 cohorts receiving the high-dose, 11 achieved the ability to sit without assistance for more than 5 s and 2 eventually were able to walk independently (Mendell et al., 2017).

In the Phase 3 SPR1NT trial (*n* = 14 presymptomatic infants with two *SMN2* copies), the results emphasized the importance of early intervention and universal newborn screening (Strauss et al., 2022). All 14 infants achieved the primary endpoint of independent sitting for at least 30 s, and 11 of 14 accomplished this endpoint within the normal developmental timeframe defined by the World Health Organization (Strauss et al., 2022).

Despite its high therapeutic efficacy, a broad spectrum of adverse events has been reported from the FDA Adverse Event Reporting System (FAERS) database (*n* = 1951), including the elevation of liver transaminases (ALT/AST), respiratory infections, metabolism and nutrition disorders, and cases of thrombotic microangiopathy (Zhang et al., 2024). Notably, treatment with Onasemnogene abeparvovec does not invariably guarantee a complete disease resolution, where infants with Type 1 SMA may still require ongoing nutritional and respiratory assistance following drug administration. Moreover, treatment outcomes appear to be influenced by age and body weight at the time of administration. Older and heavier children tend to exhibit more modest improvements in motor function, as indicative by a gain of 3.8 points on the CHOP INTEND scale in patients of greater than 2 years of age compared to a gain of 17.7 points in infants less than six months (Zhang et al., 2024; Gowda et al., 2024).

### 5.3 Risdiplam

Following Nusinersen and Onasemnogene abeparvovec, Risdiplam was the third drug introduced into clinical practice for SMA treatment and the first prescription drug for SMA in the US (Table 5), Japan and Europe (Pascual-Morena et al., 2024). It is an orally administered small molecule medication developed to modify splicing of the *SMN2* gene. Unlike other intrathecally administered ASOs, it distributes systemically to both central and peripheral tissues, thereby elevating the production of full-length SMN protein (Ratni et al., 2018; Sivaramakrishnan et al., 2017).

Mechanistically, risdiplam acts on two distinct sites on the pre-mRNA of SMN2: the 5′ splice site of intron 7 and the exonic splicing enhancer 2 located within exon 7. This interaction stabilizes the spliceosomal complex and facilitates the inclusion of exon 7 to generate full-length SMN proteins (Pascual-Morena et al., 2024; Sivaramakrishnan et al., 2017).

The clinical developmental program for risdiplam has evaluated treatment responses across different SMA phenotypes and the time of intervention. In the FIREFISH (type 1 SMA) trial, 29% of infants reached the primary endpoint of sitting without assistance for at least 5 s, and 57% accomplished a CHOP-INTEND score greater than 40 points after 12 months of treatment (Pascual-Morena et al., 2024). A comparative analysis between nusinersen and risdiplam further demonstrated that children treated with risdiplam experienced a 78% reduction of mortality and an 81% reduction in the combined risk of death or permanent ventilation (Kokaliaris et al., 2024). In the SUNFISH (Type 2 and 3 SMA) trial, treatment over 12 months resulted in a mean increase of 2.09 points in the 32-item Motor Function Measure (MFM32) and 1.73 points in the Revised Upper Limb Module (RULM) scales (Pascual-Morena et al., 2024). Consistent with observations from studies of nusinersen, the greatest clinical benefit is strongly correlated with early diagnosis and treatment. In the RAINBOWFISH (Presymptotic infants), 100% of participants achieved a CHOP-INTEND score exceeding 40 at 12 months (Pascual-Morena et al., 2024). Collectively, these studies demonstrated meaningful improvements in motor function, survival, and milestone attainment across a broad spectrum of SMA phenotypes.

Risdiplam is generally well-tolerated; however, approximately 15–20% of patients in clinical trials experience adverse events to varying extents, including fever, upper respiratory tract infections, diarrhea, and pneumonia. Real-world data from the FAERS database have also detected new potential safety risks such as cardiac arrest and raised hepatic enzymes levels (Pascual-Morena et al., 2024; Yu and Liu, 2024). Additionally, long-term observational studies of Type 2 and 3 participants and adults reveal a similar pattern of response to that observed with nusinersen. In these populations, motor function typically stabilizes or plateaus over time rather than continuing to improve (Alonge et al., 2025). In general, the efficacy and benefit of the treatment remains limited in some patients, highlighting the need for further longitudinal studies and strategies to minimize adverse events (Palacino et al., 2015).

Collectively, the clinical success of nusinersen, onasemnogen abeparvovec, and risdiplam confirms that restoring and increasing functional SMN proteins significantly improves the progression of SMA disease. However, these monotherapies have demonstrated their different clinical boundaries. While consistent benefits are observed among the three therapies when treatments are administered before or early after the onset of symptoms, patients with type II or III SMA treated later in life often achieve disease stabilization or incomplete functional recovery. Even with these clinical breakthroughs, many individuals continue to suffer from residual disabilities related to respiratory, nutritional, motor, and orthopedic aspects. These clinical trials support the conclusion that SMN restoration is an essential disease-modifying step, but it may be insufficient for patients to achieve normalization of long-term consequences in isolation. Consequently, the rationale for combinatorial approaches is an interpretive response to the common clinical limitations identified from current therapies, rather than a direct conclusion proved by established combination treatment trials. Future therapeutic strategies may focus on integrating SMN-improving agents with novel advancements targeted specifically at muscle integrity and neuromuscular junction function to more comprehensively and effectively manage the complex nature of SMA.

### 5.4 Biomarkers for SMA diagnosis, progression, and treatment monitoring

The advent of disease-modifying therapies has highlighted the need for robust, quantifiable biomarkers that reflect disease activity, predict progression, and monitor treatment response. Several molecular, biochemical, and electrophysiological markers have been investigated in SMA, and a subset has been integrated into clinical trials and real-world practice.

#### 5.4.1 SMN protein and SMN transcripts

SMN protein levels in peripheral blood mononuclear cells and skin fibroblasts correlate with SMN2 copy number and, broadly, with phenotypic severity (Lefebvre et al., 1997; Sumner et al., 2003). However, the dynamic range of SMN protein assays remains limited, and intracellular compartmentalization complicates interpretation (Coovert and Coovert, 1997). SMN2-derived full-length versus exon-7-skipped transcript ratios in whole blood have been used as pharmacodynamic endpoints for splicing modifiers such as risdiplam, providing direct evidence of target engagement (Sivaramakrishnan et al., 2017).

#### 5.4.2 Neurofilament proteins

Neurofilament light chain (NfL) in serum and cerebrospinal fluid (CSF) has emerged as a leading neurodegeneration biomarker in SMA. NfL levels are markedly elevated in untreated infants with SMA type I and decline rapidly following nusinersen or onasemnogene abeparvovec treatment, correlating with improvements in motor milestone scores (De Vivo et al., 2019; Nuzzo et al., 2023). Phosphorylated neurofilament heavy chain (pNfH) in CSF shows a similar trajectory and has been used as a secondary endpoint in clinical trials of nusinersen. These markers offer a quantifiable readout of axonal injury and neuroprotective drug effects, and they are increasingly incorporated into trial designs to enable early efficacy assessment.

#### 5.4.3 Compound muscle action potential (CMAP)

CMAP amplitude and the calculated motor unit number estimation (MUNE) reflect functional motor unit integrity. In SMA, CMAP is reduced even presymptomatically, indicating early, subclinical motor neuron dysfunction. Longitudinal CMAP measurements have shown stabilization or partial recovery following nusinersen or gene therapy (Finkel et al., 2017; Mendell et al., 2017). Because CMAP provides an electrophysiological correlate of motor unit health, it complements biochemical markers and is particularly useful for detecting treatment effects in later-onset or adult patients, in whom clinical motor scales may change slowly.

#### 5.4.4 Emerging biomarkers

MicroRNA species, including miR-146a, are being explored as immunomodulatory biomarkers that may reflect neuroinflammatory activity in SMA (Belančić et al., 2025). Additionally, functional parameters derived from iPSC-based assays, such as axonal length and SMN-dependent gene expression, are being developed as *in vitro* surrogate endpoints for drug screening. The systematic validation of these emerging biomarkers in longitudinal cohorts will be essential for their future integration into clinical practice.

In summary, while Nusinersen, Onasemnogene Abeparvovec, and Risdiplam all fundamentally aim to elevate SMN protein levels, they represent distinct pharmacological modalities with unique clinical profiles. To provide a clear and critical synthesis of their differences, Table 1 summarizes and contrasts these three approved therapies across key dimensions, including molecular mechanisms, tissue distribution, pivotal clinical trials, major safety concerns, and real-world challenges.

As synthesized in Table 7, each of the three Monotherapies possesses distinct pharmacological boundaries. While Nusinersen effectively targets the CNS, it leaves peripheral organs vulnerable to low SMN levels. In contrast, Onasemnogene abeparvovec provides systemic restoration but is limited by viral-vector immunity and a narrow therapeutic window. Risdiplam offers whole-body distribution but demands lifelong compliance. This critical mismatch between single-drug biodistribution and the multi-systemic nature of SMA forms the foundational rationale for combinatorial strategies (discussed in Section 7), shifting the paradigm from ‘SMN-monotherapy’ to ‘SMN-plus’ synergistic interventions.

**Table 7:** Pharmacological and clinical comparison of approved SMN-targeting therapies for SMA.

Comparison dimension	Nusinersen (Spinraza)	Onasemnogene abeparvovec (Zolgensma)	Risdiplam (Evrysdi)
Therapeutic class	Antisense oligonucleotide (ASO).	Gene replacement therapy (AAV9 vector).	Small molecule RNA splicing modifier.
Mechanism of action	Binds to ISS-N1 in *SMN2* pre-mRNA, promoting exon 7 inclusion.	Delivers a functional copy of human *SMN1* cDNA via non-replicating AAV9.	Modulates *SMN2* pre-mRNA splicing by binding to two distinct sites (5′ splice site and ESE).
Route and frequency	Intrathecal injection (lumbar puncture); 4 loading doses, followed by maintenance every 4 months.	Single-dose intravenous (IV) infusion.	Daily oral solution or feeding tube administration.
Biodistribution	Highly restricted to the Central Nervous System (CNS); negligible systemic exposure.	Broad systemic and CNS distribution; high tropism for the liver, motor neurons, and skeletal muscle.	Widespread systemic distribution, penetrating both the blood–brain barrier (BBB) and all peripheral tissues.
Key clinical trials	ENDEAR (Infantile-onset), CHERISH (Later-onset), NURTURE (Presymptomatic).	STR1VE (Type 1), START (Type 1), SPR1NT (Presymptomatic).	FIREFISH (Type 1), SUNFISH (Type 2/3), RAINBOWFISH (Presymptomatic).
Efficacy highlights	Significant improvement in motor milestones; reduced ventilation and mortality risk.	Rapid expression of SMN protein; enables patients to sit independently and achieve age-appropriate milestones.	Sustained motor function improvement in both infants and broad-spectrum older/adult patients.
Major safety concerns	Thrombocytopenia, coagulation abnormalities, renal toxicity, and risks associated with repeated lumbar punctures.	Acute liver injury/acute liver failure, Thrombotic Microangiopathy (TMA), and elevated troponin-I (cardiotoxicity).	Risk of retinal toxicity (monitored in trials), potential reproductive toxicity (male fertility concerns).
Real-world challenges	High treatment burden; technical difficulty in patients with severe scoliosis.	Immune response against AAV9 vector (pre-existing antibodies rule out eligibility); exorbitant single-dose cost.	Strict requirement for daily adherence; potential drug–drug interactions due to systemic exposure.

## 6 Emerging and adjunctive therapeutic strategies

In addition to the three approved SMN-targeting therapies, a growing number of non-SMN strategies are being investigated to address the multifactorial pathology of SMA. These approaches target distinct but interconnected pathways, including skeletal muscle preservation, neuromuscular junction stability, neuroinflammation, cellular stress responses, and next-generation vector technologies. While none of these have yet received regulatory approval for SMA, several have advanced to clinical trials, and all reflect the evolving recognition that SMN restoration alone may not suffice for complete disease reversal. The following subsections summarize the most clinically and translationally advanced of these adjunctive strategies.

### 6.1 Apitegromab

Apitegromab (SRK-015) is a monoclonal antibody that inhibits latent myostatin activation, a mechanism aimed at promoting skeletal muscle growth and hypertrophy. Myostatin is a negative regulator of muscle mass, and its inhibition represents a promising non-SMN-dependent strategy. In the TOPAZ Phase 2 study, apitegromab demonstrated favorable safety and efficacy, particularly when used in conjunction with nusinersen, with improvements in HFMSE scores among ambulatory and non-ambulatory Type II and III SMA patients (Barrett et al., 2021; Day et al., 2022). Apitegromab may be most effective as an adjunct therapy rather than monotherapy, enhancing the functional capacity of existing SMN-based treatments. Ongoing investigations are focused on its long-term safety and efficacy in pediatric populations.

### 6.2 Reldesemtiv

Reldesemtiv is a fast skeletal muscle troponin activator (FSTA) that increases the calcium sensitivity of the troponin complex in fast-twitch muscle fibers, thereby enhancing muscle contractility without increasing intracellular calcium concentration. In a Phase 2 trial, patients receiving 450 mg reldesemtiv twice daily showed significant gains in six-minute walk distance and expiratory pressure (Rudnicki et al., 2021). However, no durable improvement in overall motor function was observed, and the trial failed to meet long-term efficacy endpoints. The program was discontinued in 2021 due to strategic reprioritization by the sponsor. Reldesemtiv highlights the challenges of achieving sustained functional gains solely through symptomatic muscle enhancement.

### 6.3 BVS857

BVS857 is an engineered IGF-1 mimetic designed to stimulate muscle growth by promoting myoblast proliferation and protein synthesis. Though early studies in neuromuscular disorders indicated preservation of muscle volume, a Phase 2 trial in SBMA showed no significant benefit in muscle strength. The high incidence of immunogenicity, with over 70% of patients developing anti-drug antibodies—some targeting endogenous IGF-1—raised safety concerns (Grunseich et al., 2018). The development of BVS857 was terminated, underscoring the limitations of systemic growth factor analogs in chronic neuromuscular disease contexts. Future directions may involve localized delivery or gene-regulated expression systems to mitigate systemic risks.

### 6.4 Neuroinflammation

Neuroinflammation is increasingly recognized as a contributor to SMA pathophysiology. Elevated IL-6 and TNF-*α* levels, derived from activated microglia and astrocytes, have been linked to motor neuron degeneration. These cytokines amplify neurodegenerative cascades via NF-κB signaling. SMN-enhancing agents such as nusinersen have been shown to reduce microglial activation and suppress pro-inflammatory cytokines (Nuzzo et al., 2023). Additionally, microRNA candidates like miR-146a act as endogenous repressors of inflammatory signaling, offering potential for fine-tuned immunomodulation (Belančić et al., 2025). However, the complexity of CNS immune networks and risk of off-target effects necessitate further study before clinical translation.

### 6.5 Autophagy and ER stress

Autophagy and ER stress pathways play key roles in maintaining neuronal proteostasis. In SMA, SMN deficiency can impair the unfolded protein response (UPR), leading to ER stress and apoptosis. Concurrently, dysregulation of mTOR suppresses autophagy, compromising the cell’s ability to clear damaged proteins and organelles. Small molecules such as salubrinal (eIF2α phosphorylation modulator) and Ceapins (ATF6 branch inhibitor) have shown preclinical efficacy in modulating UPR and reducing neuronal death (Almanza et al., 2019; Nguyen et al., 2024). These findings support the therapeutic potential of targeting cellular stress responses, although no candidate has advanced to clinical evaluation in SMA as of 2025.

### 6.6 AAV-based gene delivery

AAV-based gene delivery remains foundational in SMA therapy, yet safety concerns related to high vector doses and limitations on repeat dosing persist. Adverse events including hepatotoxicity and immune-mediated responses have prompted the development of next-generation capsids with improved CNS tropism, enhanced transduction efficiency, and reduced immunogenicity. For patients with non-CNS-dominant SMA or those unresponsive to existing therapies, novel AAV variants may enable targeted delivery to peripheral tissues or circumvent pre-existing neutralizing antibodies. Preclinical data are encouraging, but translation to human studies remains ongoing (Wang et al., 2024; Xu et al., 2024).

Together, these non-SMN-targeted strategies represent the next logical step in SMA therapeutics, which we believe must be developed in concert with—not as replacements for—SMN restoration.

## 7 Newborn screening for SMN mutations

### 7.1 Pre-symptomatic screening

SMA screening is crucial when effective therapies are available, as the therapeutic efficacy may be compromised if the diagnosis is delayed. The efficacy of treatments for presymptomatic patients has been good in clinical trials of Nusinersen and Onasemnogene abeparvovec (De Vivo et al., 2019). For example, patients predicted to have SMA type II could walk independently if they were treated before symptoms developed (De Vivo et al., 2019). Based on these results, SMA screenings have been increasingly implemented worldwide (Dangouloff et al., 2020; Dangouloff et al., 2021). Initiating treatment in the presymptomatic stage and maximizing therapeutic efficacy are essential and rely on successful screening programs.

### 7.2 Prevention of delayed diagnosis of SMA

Presymptomatic treatment is impossible if diagnosis is delayed. Newborn screening can help to prevent such delays and allow treatment to be given in time, possibly leading to improved motor function recovery (Noguchi et al., 2022). Infants with hypotonia and/or respiratory problems should have SMA considered as a diagnosis.

### 7.3 Strategies for SMA treatments

A report titled “Treatment Algorithm for Infants with SMA Detected by Newborn Screening” was published in 2018 (Glascock et al., 2018). Both presymptomatic and symptomatic SMA can be diagnosed by newborn screening and treated accordingly. The treatment algorithm was based on the SMN2 copy number rather than symptoms. For type 0 SMA with only one copy of SMN2 with any symptoms, the decision of whether to initiate treatment should be made by physicians. Type I SMA with two or three copies of SMN2 are expected to develop symptoms, therefore early initiation of treatment is needed. Similarly, for type II or III with three copies of SMN2, early initiation of treatment is needed. For type III or IV SMA with four or more copies of SMN2, treatment may only be needed if signs of SMA develop later in life. This algorithm was changed in the revised version in 2020, with the authors recommending that patients be treated as soon as possible regardless of their SMN2 copy number, including for those with four or more SMN2 copies. A study in Germany found that among 278,970 infants screened for SMA, 40% had four or more SMN2 copies (Müller-Felber et al., 2020).

### 7.4 Challenges for SMA screening

Despite the availability of new drugs for SMA treatment, only a limited number of countries actually use these drugs for SMA patients (Dangouloff et al., 2021). The high cost of these new drugs makes their use impractical in many countries. The cost – benefit data for the implementation of newborn screening and treatment also vary between countries (Dangouloff et al., 2021; Kariyawasam et al., 2021). In Japan, for example, there is a lack of knowledge in the public about SMA screening and new treatments (Lee et al., 2021). Raising public awareness of SMAs would be the first step forward in many countries.

A particularly challenging ethical and clinical dilemma has emerged with the increasing implementation of newborn screening programs: how to manage infants identified as having four or more copies of *SMN2*. This subgroup constitutes a substantial proportion of screen-positive cases—in the German newborn screening pilot project encompassing 278,970 infants, 40% of the 38 confirmed SMA cases carried four or more *SMN2* copies (Müller-Felber et al., 2020). While the 2020 revised treatment algorithm moved toward recommending early intervention regardless of *SMN2* copy number (Glascock et al., 2018), this recommendation remains controversial, and there is still no worldwide consensus on presymptomatic treatment for patients with four or more *SMN2* copies (Ricci et al., 2023).

The controversy stems from the extraordinary phenotypic heterogeneity observed in this population. While four *SMN2* copies are generally associated with milder SMA types (III or IV) and later onset, the clinical spectrum is remarkably broad. In a nationwide Italian cohort of 169 patients with four *SMN2* copies, phenotypes ranged from type II (8 patients) to type III (145 patients) and type IV (8 patients), with 6 patients remaining presymptomatic and 2 asymptomatic adults identified through familial cases (Ricci et al., 2023). Importantly, cross-sectional functional data demonstrated a progressive decline with increasing age: over 35% of type III patients and 25% of type IV patients lost ambulation, with a mean age of loss at 26.8 years (Ricci et al., 2023). A retrospective analysis of 268 untreated patients from the SMArtCARE registry in Germany, Austria, and Switzerland further revealed that the median age of disease onset was 3.0 years, and 55% of patients experienced symptoms before the age of 36 months (Vill et al., 2024). Moreover, 3% never learned to sit unaided, 13% never gained the ability to walk independently, and 33% of ambulatory patients lost this ability during the course of the disease; 43% developed scoliosis, and 6.3% required non-invasive ventilation (Vill et al., 2024). These findings challenge the assumption that four-copy patients are uniformly mild and underscore that a significant proportion develop irreversible symptoms within the first few years of life.

The effectiveness of newborn screening for SMA has been rigorously demonstrated in a nonrandomized controlled trial using data from the SMArtCARE registry, which compared 44 children diagnosed through newborn screening with 190 children diagnosed after clinical symptom onset. Children in the screening cohort started treatment at a mean age of 1.3 months compared with 10.7 months in the clinical diagnosis cohort; 90.9% of screened children gained the ability to sit independently versus 74.2% in the unscreened cohort, and for independent ambulation, the rates were 63.6% versus 14.7%, respectively (Schwartz et al., 2024). These data provide compelling evidence that early detection through newborn screening dramatically improves functional outcomes. However, they also intensify the ethical dilemma for four-copy infants: while screening clearly benefits those who would otherwise present symptomatically, it also identifies a subset of infants who might never develop clinically meaningful disease, yet for whom the option of watchful waiting carries the tangible risk of irreversible motor neuron loss should symptoms eventually emerge.

The ethical dimensions of this dilemma are multifaceted. *First*, the long-term safety profile of onasemnogene abeparvovec—including risks of hepatotoxicity, thrombotic microangiopathy, and the uncertain durability of episomal vector expression in dividing cells—must be weighed against the possibility that some four-copy infants might remain asymptomatic for decades (Zhang et al., 2024). *Second*, the extraordinary financial cost of these therapies places substantial burden on healthcare systems and raises questions of resource allocation, particularly when treating patients who may derive uncertain absolute benefit. *Third*, the psychological impact on families—who must make rapid, high-stakes decisions under extreme time pressure in the neonatal period—is considerable, with some families in the German screening program even lost to follow-up to avoid the psychological stress associated with medical appointments (Müller-Felber et al., 2020).

Adding to this complexity, a recent study from German screening projects found that among 21 patients with four *SMN2* copies identified over a three-year period, eight who underwent presymptomatic therapy between 3 and 36 months of age had no definite disease symptoms to date; however, five of the other seven patients who followed a strict wait-and-see strategy showed clinical or electrophysiological disease onset between 1.5 and 4 years of age, and in two of them, complete recovery was not achieved despite immediate treatment after symptom onset (Blaschek et al., 2022). These data argue that a wait-and-see strategy carries tangible risks of irreversible motor neuron loss, yet they also highlight that the optimal timing of intervention remains uncertain.

We posit that the field urgently needs: (i) better risk-stratification tools, including validated biomarkers (e.g., neurofilament light chain) and additional genetic modifiers (e.g., *PLS3*, *NCALD*), to identify which four-copy infants are genuinely at risk of early symptom development; (ii) long-term observational registries to track the natural history of treated versus untreated four-copy infants; and (iii) a shared decision-making framework that transparently communicates the uncertainties and explicitly offers a monitored observational pathway as a legitimate alternative to immediate intervention (Blaschek et al., 2022). Until such evidence and frameworks are established, the recommendation to treat all screen-positive infants regardless of *SMN2* copy number should be regarded as an area of active debate—one that will require ongoing input from clinicians, researchers, families, and ethicists alike.

## 8 Discussion

### 8.1 A critical perspective on current SMA therapeutics

The approval of three SMN-targeted therapies has transformed SMA from a fatal disorder into a treatable condition, but the convergence of clinical evidence now delineates the boundaries of SMN-only approaches. Presymptomatic treatment yields the greatest benefit, yet even early-treated patients may retain residual motor deficits, scoliosis, and systemic complications (Audic et al., 2024; Mendell et al., 2021). In later-onset and adult SMA, functional gains are often modest, reflecting the limited capacity of postnatal SMN restoration to reverse chronically compromised motor units (Alonge et al., 2025; Moshe-Lilie et al., 2020). We therefore take the position that SMN-based therapy serves as the essential foundation, not the final destination, and that future progress depends on combinatorial strategies pairing SMN restoration with agents that directly protect the neuromuscular axis, enhance muscle function, or suppress pathogenic secondary cascades. This perspective informs our critical comparison of treatment modalities below.

### 8.2 Current challenges with treatment choices and switching

New treatments can cause new challenges, such as how to choose the right treatment for parents of infants who are first diagnosed with SMA. For those SMA patients who have received treatment with one of the three drugs, if the treatment is not as effective as they expected, deciding whether to switch drugs can be problematic. Additionally, patients who missed the optimal window for treatment may be concerned as the efficacy of the new drugs is limited outside the ideal treatment timeframe (Day et al., 2022; Moshe-Lilie et al., 2020). This therapeutic window limitation directly supports our view that monotherapy with SMN-enhancing drugs is insufficient in late-diagnosed individuals and strengthens the case for combination approaches targeting muscle and the neuromuscular junction.

### 8.3 Therapeutic options for adults with SMA

When Nusinersen became available in Italy, patients with SMA were referred to tertiary centers for the new treatment (Sansone et al., 2021). A similar situation was observed in Japan where an increased number of SMA patients were diagnosed on Shikoku Island when Nusinersen became available for patients at these sites (Okamoto et al., 2022).

The high expectations of Nusinersen from patients and families did lead to some disappointment and frustration, which may have caused unnecessary discontinuation of treatment (Agosto et al., 2023). Some patients found it less effective or experienced side effects. The distress or anxiety may be caused by the burdensome nature of the intrathecal injection procedure and the lack of perceived improvement (Agosto et al., 2021). From our perspective, these qualitative experiences highlight that drug selection cannot be based solely on SMN restoration efficacy; factors such as administration burden, systemic versus CNS-limited distribution, and patient age-dependent responsiveness must be integrated into the treatment decision hierarchy.

Consequently, some patients were switched from Nusinersen to Risdiplam (Agosto et al., 2023). Conversely, some patients switched from Risdiplam to Nusinersen due to side effects such as leukocytoclastic vasculitis. In adult SMA patients, the switch from Nusinersen to Risdiplam was likely due to perceived effectiveness and financial cost whereas the switch from Risdiplam to Nusinersen was more likely related to the side effects of Risdiplam. Due to the lack of consensus guidelines, the choice of treatment and/or switch for patients are made individually (Ramos-Platt et al., 2022). It is up to each clinician to make a decision for their patient based on the individual situation.

However, it must be explicitly stated as a critical knowledge gap that there are currently no formalized, globally accepted clinical consensus guidelines or standardized international protocols to govern treatment switching in SMA patients. The three studies examined herein do not directly address switching strategies: Severa et al. focused on risdiplam’s effects in a small cohort of six adult patients, noting that two had previously discontinued nusinersen due to side effects (Andrés-Benito et al., 2024); Andrés-Benito et al. evaluated neurodegeneration biomarkers in 31 adult SMA patients treated with nusinersen, finding that clinical improvements were statistically significant only in walkers (Andrés-Benito et al., 2024); and the phase 3 SAPPHIRE trial (Crawford et al., 2025) assessed the novel agent apitegromab in nonambulatory type 2 or 3 SMA patients receiving nusinersen or risdiplam (Severa et al., 2024). The absence of switch-specific evidence in these trials underscores the paucity of dedicated data in this domain. Consequently, switching decisions in real-world practice remain largely empirical and are tailored on a highly individualized basis.

Clinical data regarding long-term safety, optimal washout periods, and potential synergistic risks of transitioning between distinct modalities—such as from an intrathecal antisense oligonucleotide (ASO) to an oral small molecule—remain scarce. In the absence of direct comparative evidence, currently observed empirical switching is primarily driven by three real-world factors:

1. Technical and clinical barriers: Severe scoliosis, spinal deformities, or prior spinal fusion surgery that render repeated lumbar punctures for nusinersen technically impossible or highly distressing for the patient.
2. Suboptimal efficacy or therapeutic plateau: Patients who experience a perceived stagnation or decline in motor function after long-term monotherapy.
3. Patient preference and compliance: The substantial logistical advantage of a daily oral solution (risdiplam) over clinic-bound, invasive intrathecal injections, which significantly improves quality of life for adult patients.

Given the absence of direct comparative or switch-related data in the pivotal trials available to date—including the SAPPHIRE trial (Crawford et al., 2025), which evaluated a muscle-targeting therapy with a distinct mechanism of action (Severa et al., 2024) rather than ASO-to-small-molecule transition—any switch decision must be grounded in a multidisciplinary assessment that integrates individual motor function trajectories, treatment burden tolerance, and patient-reported outcomes. Until dedicated prospective studies on switching protocols are conducted, clinicians are advised to follow the general principle of individualized risk–benefit evaluation, as endorsed by current consensus statements from major neuromuscular societies.

### 8.4 Technical barriers on durability and delivery

Given the limited long-term efficacy of current treatment and the technical barriers faced to reach a “definitive cure,” durability of therapeutic benefit has become a major challenge. For example, the persistence of AAV vectors in gene replacement therapy is not guaranteed as they remain episomal and do not integrate into the host genome. This is particularly variable in young children, whose cells keep dividing and the vector DNA gets diluted over time. Additionally, high-dose viral delivery raises real safety concerns, with reports of thrombotic microangiopathy being common.

These barriers have pushed researchers to search for new appraoches that focus on stable genomic integration. One promising direction is to combine existing therapies with muscle-targeted approaches and genome editing, which could potentially fill in the gaps in long-term durability. The progress toward the refinement of these technologies would also face additional set of difficulties associated with safety, complexity, and accuracy.

### 8.5 Future prospects for SMA therapies

Current standard care for SMA emphasizes early diagnosis via newborn screening and timely initiation of SMN-targeted treatments, which are now widely available and accessible to affected individuals. However, a significant unmet need persists as existing treatments cannot restore the damage that has already occurred. This limitation has prompted the exploration of combination therapy, marking a strategic approach to surpass the boundaries of single agent SMN-targeted therapies and tackle with the evolving and intricate pathophysiology of SMA.

#### 8.5.1 Precision medicine and genotype-guided treatment

SMN2 copy number remains the primary genetic modifier guiding treatment urgency; however, genotype–phenotype correlations are imperfect. For instance, newborn screening increasingly identifies infants with four SMN2 copies, for whom the optimal timing of treatment remains debated because some may remain asymptomatic for years (Müller-Felber et al., 2020). Incorporating additional genetic modifiers such as PLS3 and NCALD, as well as emerging biomarkers, into risk-stratification algorithms may refine treatment decisions (Riessland et al., 2017; Oprea et al., 2008). A precision medicine framework that integrates genetic, biochemical, and electrophysiological data could ultimately enable more individualized therapeutic approaches.

#### 8.5.2 Rationale for combination therapy

SMN-dependent therapies largely restore SMN levels in the CNS and peripheral tissues, but have limited direct impact on intrinsic muscle pathology, neuromuscular junction dysfunction, and neuroinflammation. This provides a strong biological rationale for combining SMN restoration with agents that target downstream pathways. Pharmacological inhibitors of myostatin (e.g., apitegromab) have shown promising results in phase 2 trials when added to nusinersen, supporting the “SMN plus muscle” concept (Day et al., 2022). Preclinical studies also indicate that combining nusinersen with small molecules that independently enhance SMN2 expression, such as histone deacetylase inhibitors (e.g., valproic acid), may produce additive effects, although clinical validation is lacking (Brichta et al., 2003; Sumner et al., 2003). Ongoing and future clinical trials will need to define optimal combinations, sequencing, and patient populations.

#### 8.5.3 Next-generation gene editing and delivery

Although onasemnogene abeparvovec provides durable SMN expression in post-mitotic cells, its episomal vector genome may be diluted during cell division, potentially limiting life-long efficacy. The Gene-DUET strategy, which couples AAV-mediated SMN gene supplementation with CRISPR-Cas9-based homology-independent targeted integration (HITI), has been shown to achieve sustained genomic integration and improved long-term survival in SMA mice compared with gene supplementation alone (Hatanaka et al., 2024). This approach overcomes the reliance on homology-directed repair, which is inefficient in non-dividing motor neurons. Beyond HITI, emerging technologies such as base editing and prime editing offer the potential to directly correct the C-to-T mutation in SMN2 without inducing double-strand breaks, representing a next generation of gene therapy candidates for SMA (Wang et al., 2024; Xu et al., 2024).

#### 8.5.4 Biomarker-driven trial design

The integration of validated biomarkers—including neurofilament light chain (NfL), phosphorylated neurofilament heavy chain (pNfH), and compound muscle action potential (CMAP)—into future clinical trials will be essential for early assessment of treatment response, dose optimization, and patient stratification. Blood-based pharmacodynamic readouts such as NfL can reflect neuronal damage and drug activity, enabling more efficient trial designs and potentially facilitating regulatory decision-making. A dedicated discussion of SMA biomarkers is provided in the newly added Section 5.4.

This therapeutic strategy has been validated in cell and animal studies. The next step would be to confirm the efficacy and safety of these agents in clinical trials before they are applied to SMA therapies.

## 9 Conclusion

The era of early diagnosis and treatment with effective drugs has set a new standard of care for SMA. Nusinersen, onasemnogene abeparvovec, and risdiplam each provide substantial, mechanism-specific benefits, yet their clinical impact is greatest when initiated presymptomatically and diminishes with diagnostic delay. This therapeutic-window dependency, together with the persistence of residual motor deficits and systemic complications even in early-treated individuals, indicates that SMN restoration alone is insufficient to achieve a complete cure.

The key conclusions of this review are threefold. First, newborn screening and presymptomatic intervention remain the most powerful determinants of treatment success, underscoring the need for universal implementation and public education. Second, the growing recognition that SMA pathophysiology extends beyond motor neuron cell bodies to the neuromuscular junction, skeletal muscle, and systemic organs provides a biological rationale for combinatorial strategies that pair SMN-enhancing drugs with agents targeting muscle growth, cytoskeletal integrity, and neuroinflammation. Third, validated biomarkers—including neurofilament light chain, phosphorylated neurofilament heavy chain, and compound muscle action potential—are now being integrated into clinical trials and hold promise for guiding personalized treatment decisions.

Reflecting on the history of SMA, the progress achieved in diagnosis and treatment is remarkable; however, it is only the beginning. For the substantial population of older children and adults living with SMA, and for infants who do not achieve expected milestones despite early treatment, the development of SMN-independent and combinatorial approaches remains an urgent priority. We anticipate that the next decade will see the maturation of precision medicine frameworks that integrate genetic risk stratification, biomarker monitoring, and sequential or concurrent multi-target therapies, moving the field closer to a definitive cure for all SMA patients.
